# Vibration Separation Methodology Compensated by Time-Varying Transfer Function for Fault Diagnosis of Non-Hunting Tooth Planetary Gearbox

**DOI:** 10.3390/s22020557

**Published:** 2022-01-12

**Authors:** Shuiguang Tong, Junjie Li, Feiyun Cong, Zilong Fu, Zheming Tong

**Affiliations:** 1 The State Key Laboratory of Fluid Power and Mechatronic Systems, Zhejiang University, No. 38 Zheda Road, Hangzhou 310027, China; cetongsg@zju.edu.cn (S.T.); 11825060@zju.edu.cn (J.L.); fycong@zju.edu.cn (F.C.); 11825064@zju.edu.cn (Z.F.); 2 School of Mechanical Engineering, Zhejiang University, No. 38 Zheda Road, Hangzhou 310027, China

**Keywords:** vibration separation, time-varying transfer function, non-hunting ratio, planetary gearbox, fault diagnosis

## Abstract

Due to planetary movement of planet gears, the vibration signal perceived by a stationary sensor is modulated and difficult to diagnose. This paper proposed a vibration separation methodology compensated by a time-varying transfer function (TVTF-VS), which is a further development of the vibration separation (VS) method in the diagnosis of non-hunting tooth planetary gearboxes. On the basis of VS, multi-teeth VS is proposed to extract and synthesize the meshing signal of a planet gear using a single transducer. Considering the movement regularity of a planetary gearbox, the time-varying transfer function (TVTF) is represented by a generalized expression. The TVTF is constructed using a segment of healthy signal and an evaluation indicator is established to optimize the parameters of the TVTF. The constructed TVTF is applied to overcome the amplitude modulation effect and highlight fault characteristics. After that, experiments with baseline, pitting, and compound localized faults planet gears were conducted on a non-hunting tooth planetary gearbox test rig, respectively. The results demonstrate that incipient failure on a planet gear can be detected effectively, and relative location of the local faults can be determined accurately.

## 1. Introduction

Planetary gear transmissions are widely used in industrial and military applications, such as wind turbine [1] and helicopter main rotor [2], mainly because they can provide large torque and speed ratio within a compact space. Since planetary gearboxes are usually working under heavy load and tough conditions, the gears are prone to failure, resulting in tooth pitting, spalling, and even cracking. The faults may further induce shutdown or even catastrophe, which causes economic loss or security incidents. As a result, the condition monitoring and fault diagnosis of a planetary gearbox is vital important.

In the past few decades, vibration-based analysis techniques have been developed successfully to detect weak fault characteristics and incipient damage in fixed axial gearboxes, but most of these techniques cannot be applied to a planetary gearbox directly. Researchers modified or adapted some techniques to diagnose planetary gearboxes. Kong et al. [3] established an adaptive empirical wavelet transform framework for fault-related mode extraction for planet gear. Mauricio et al. [4] implemented an improved envelope spectrum via feature optimization-gram for diagnosis for the planet bearings. Feng et al. [5] used ensemble empirical mode decomposition (EEMD) and energy separation to diagnose the sun gear of a planetary gearbox. Jiang et al. [6] proposed a denoising method based on adaptive Morlet wavelet and singular value decomposition to extract the fault feature in wind turbine vibration signals. Smith et al. [7] used a simple spectral kurtosis-based approach to select the best demodulation band and extracted a planet bearing fault-related characteristic. He et al. [8] proposed a feature extraction method based on sparse decomposition and order tracking to achieve the health monitoring of a non-stationary planetary gearbox.

Although plenty of researchers have shown that vibration-based analysis techniques are effective in diagnosing gearboxes, the aforementioned conventional signal processing methods usually require a high degree of expertise [9]. The requirement of experience limits the promotion and development of these methods. In order to address this issue, intelligent diagnosis methods are introduced to diagnose the planetary gearbox and gradually become a new trend and hot topic. The deep learning algorithm [10,11,12] treats the planetary gearbox as a black box and performs well; although, it needs a large amount of data.

To figure out more about the vibration mechanism of the planetary gearbox, a lot of dynamic-based or phenomenon-based models have been established to reveal the vibration characteristic and modulation sidebands generation mechanism. Inalpolat et al. The authors in [13] proposed a mathematical model to describe the mechanism leading to modulation sidebands in five distinct groups, and planetary movement that is firstly represented by a Hanning function. Lei et al. [14] established phenomenon models of vibration signals and deduced the spectral structures for condition monitoring of the epicyclic gearbox, which also considers the time-varying transfer paths as a Hanning function. Park et al. [15] built a lumped parametric model and applied a gear mesh stiffness and transmission error-based technique to fault diagnosis of a planetary gearbox. Feng et al. [16] gave the signal model of a planetary gearbox and summarized the spectral characteristics considering both the amplitude modulation and the frequency modulation effects due to gear damage and the periodically time variant working condition. The effect of the vibration transfer path is depicted by a trigonometric function in their model. After that, a planet-bearing vibration signal model developed by the explicit equations of Fourier spectrum was derived [17]. Liu et al. [18] proposed a non-linear dynamic-based vibration model, and one comprehensive modeling method for transmission path effect with two different window functions was given. Li et al. [19] built a vibration signal model to analyze modulation sidebands based on the transfer function and measuring-direction projection function to identify the distributed defects on floating sun gear. The transfer path function is expressed by a periodic function and further represented by Fourier series in their model.

According to the vibration models built above, it can be summarized that the description of the time varying transfer path is vital important. The periodic time-varying transfer path leads to complicated amplitude modulation, resulting in amplitude envelope and modulation sidebands. The vibration signal received by a stationary sensor is modulated by the effect of the time-varying transfer path. As a result, the modulated signal may mask the incipient fault characteristic or trigger a false alarm when using traditional signal processing methodologies and a health indicator. It is necessary to establish reasonable TVTF to reveal regularity of movement and overcome amplitude modulation effects.

When analyzing a planetary gearbox vibration signal, it would be easier if the planetary gearbox signal could be converted into the fixed-axial gearbox signal. Based on this idea, McFaden et al. [20,21] firstly presented a windowing and mapping strategy to apply time domain averaging (TSA) to the vibration of planetary gearbox, which is called vibration separation (VS). Samuel et al. [22] proposed a constrained adaptive lifting (CAL) algorithm for detecting gear faults in planetary gearbox using signal generated from the VS algorithm. Lewicki et al. [23] performed thorough studies to demonstrate the capability of VS to detect different planet gear and bearing faults. Zhang et al. [24,25,26,27,28] proposed a fault diagnosis method for sun gear faults based on the continuous VS method and minimum entropy deconvolution. Guo et al. [29,30] proposed an improvement on synthesizing the artificial gear vibration in VS and combined VS and narrowband demodulation for tooth fault detection. Liang et al. [31] conducted the VS to achieve the fault detection on planet gear and tested the VS with both simulated and experimental signal. Compared with other vibration-based analysis techniques, the VS method is able to show faults more intuitively as a time domain analysis method, and it has been shown to diagnose the planetary gearbox effectively.

Due to the gearbox parameters and motion regularity, the complete VS signal can only be extracted from planetary gearboxes with a hunting ratio between the planet and ring gears, and the sun and ring gears [32]. Hunting ratio tooth planetary gearboxes show better performance in vibration and noise, thereby reducing wear and extending service life. Hence, many gearboxes are designed with a hunting ratio between meshing gears; however, non-hunting tooth gear sets are still widely in service in practical applications currently [33,34,35] because traditional VS is aimed at extracting all the teeth meshing signal of the target gear. However, traditional VS cannot acquire all teeth signal in a non-hunting gear set. As a result, the traditional VS using a single transducer will be disabled when applied to the non-hunting tooth planetary gear sets. It is meaningful to search effective methods for diagnosis of a planetary gearbox with a non-hunting ratio tooth. Samuel et al. [32] proposed the extension of the technique using multiple transducers in order to overcome the limitations of the single transducer VS technique. However, this method is too costly and inconvenient to implement in the filed application because multiple transducers are required. Few studies have researched how to apply VS to the diagnosis of non-hunting tooth planetary gearbox.

To settle this problem, a vibration separation methodology compensated by a time-varying transfer function (TVTF-VS) is proposed in this paper to diagnose the non-hunting tooth planetary gearbox using a single transducer. In our methodology, the multi-teeth vibration separation (multi-teeth VS) algorithm is proposed to extract and synthesize the meshing signal of an individual planet gear in a non-hunting tooth planetary gearbox. Subsequently, TVTF is constructed using a segment of healthy signal to overcome the amplitude modulation effect existing in the multi-teeth VS waveform. After compensation of the TVTF, the resultant TVTF-VS waveform is obtained. Finally, the diagnosis conclusions are drown based on the resultant TVTF-VS. The main innovation of the proposed methodology is that the TVTF-VS algorithm can be applied to non-hunting tooth planetary gearboxes with only one transducer used. Two sets of planet gear fault-seeded experiments, pitting and compound localized faults, are conducted to verify the performance of proposed methodology. According to the experimental results, the TVTF-VS methodology proved to be effective in planet gear fault detection and location.

The organization of this paper is as follows: Section 2 introduces the framework and implementation of multi-teeth VS. Subsequently, Section 3 analyzes the vibration transfer path of planetary gearbox and shows the construction of TVTF. After that, Section 4 proposes the TVTF-VS methodology and exhibits the technical route of the proposed TVTF-VS. In Section 5, the methodology is validated using two experimental demonstrations. Finally, Section 6 draws the conclusion and summarizes this paper.

## 2. Multi-Teeth Vibration Separation

### 2.1. Framework of Multi-Teeth VS

**Figure 1:**
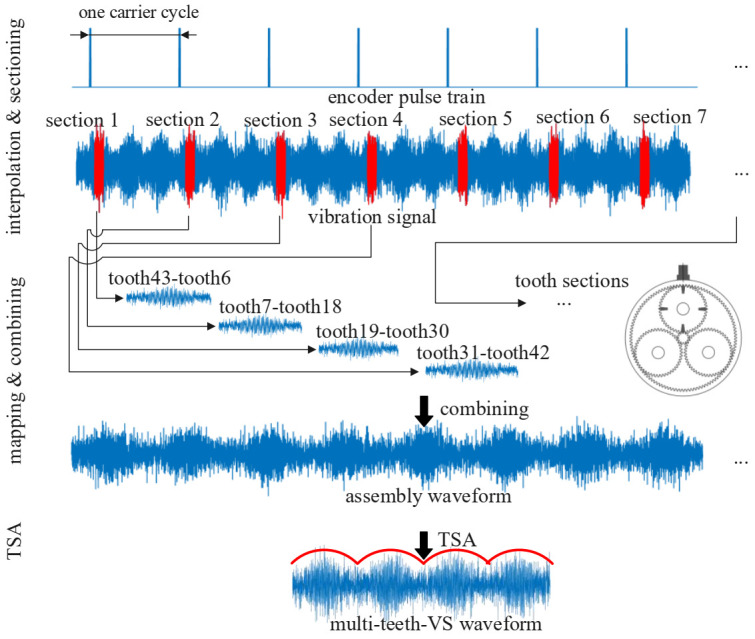
Schematic diagram of multi-teeth VS.

In order to apply the VS method to the non-hunting tooth planetary gearbox, this paper proposes a modified VS methodology called multi-teeth VS. The multi-teeth VS mainly contains 3 steps and the schematic of multi-teeth VS is illustrated in Figure 1.

Step 1: Interpolating and multi-teeth sectioning. The encoder pulse train and vibration signal are collected simultaneously. Then, vibration signal is partitioned into blocks every one carrier cycle. It should be noted that the location of blocks is decided by the encoder pulse train during the partition process. Since carrier rotates once, $N_{p}$ planet gears pass by the sensor successively, one slice (called section in this paper) in a block can be extracted to represent vibration signal when planet gear $P_{i}$ is just under the sensor. Each section contains information about several planet gear teeth meshing with the ring gear before and after the acquisition point. For example, Section 2 contains meshing information about tooth 7 to tooth 18 on planet gear as shown in Figure 1. The location and the length of sections will be explained in Section 2.3.

Step 2: Mapping and combining. After extracting the sections from vibration signal, the sections are mapped into assembly matrix according to the teeth meshing sequence, which will be introduced in Section 2.2. It should be noted that each section extracted in multi-teeth VS is responding to $M_{t}=\frac{Z_{p}}{n_{rest,p}}$ teeth. Then the meshing signal of one planet gear is acquired for several revolutions.

Step 3: TSA. The TSA is applied to the meshing signal of several revolutions for $M_{e}$ times and the $M_{e}$ is usually more than 10. Since the first and the last cycle is usually incomplete, and they are discarded, the length of vibration signal collected is at least $n=M_{e}\cdot n_{rest,p}+2$ cycles. After averaging procedure, meshing signal of one revolution is obtained.

### 2.2. Teeth Meshing Sequence

The teeth meshing sequence of planetary gearbox follows some certain regularities. It is noted that the stationary sensor is usually fixed on the ring gear. When an individual planet gear passes by a sensor, one particular tooth on the planet gear meshes with the ring gear under sensor. At the end of next revolution of carrier, another tooth on the planet gear is in mesh with the ring gear. After several rotations of the carrier, the same tooth on the planet gear will mesh with the ring gear at the same position relative to the ring gear. Similarly for the sun gear, the same tooth on the sun gear will mesh with one particular planer gear aligning to the sensor (the same position relative to the ring gear) after several rotations of carrier.

In other words, the teeth meshing sequence will repeat periodically after several rotations of the carrier. The least number of carrier rotations can be modeled by:

(1) $$n_{rest,g}=\frac{LCM(N_{g},N_{r})}{N_{r}}$$

where *LCM* denotes the least common multiple, $Z_{r}$ is the teeth number of ring gear, $Z_{g}$ is the teeth number of interesting gear (planet gear or sun gear). It should be noted that if the planetary gearbox has a hunting ratio between the ring gear the and planet gear (the same as the sun gear), the $n_{rest,p}=Z_{p}$, each tooth on the planet gear will mesh with the ring gear at the same position in $n_{rest}$ carrier rotations. Moreover, for the planetary gearbox with a non-hunting ratio between the ring gear and the planetary gear, only partial teeth on the planet gear participate the periodic meshing sequence. The order of the teeth meshing sequence can be given by:

(2) $$P_{n,g}=\mathrm{MOD}(nN_{r},N_{g})+1$$

where $P_{n,g}$ is the teeth order of the interesting gear meshing with the ring gear at the same position when the carrier rotates nth, the n=0,1,2…, n=0 means the initial teeth order, MOD is modulo operation. For example, Table 1 lists two types of planetary gearboxes of different parameters. The teeth meshing sequences of the planet gear with the same position relative to the ring gear in each gearbox are shown in Table 2 and Table 3, respectively. As Table 2 shows, all the 22 teeth on planet gear mesh with the ring gear at the same position successively for $n_{rest,p}=22$ revolutions of carrier. However, as Table 3 shows, only 4 teeth on planet gear with total 48 teeth participate in the meshing sequence and it repeats for $n_{rest,p}=4$ revolutions of carrier rotation. Hence, type 1 and type 2 planetary gearboxes are referred to as the hunting ratio planetary gearbox and non-hunting ratio planetary gearbox.

### 2.3. The Location and Length of Sections

The extracted sections should contain more information about the objective planet gear. There are two methods to determine the location of sections. The first is tracking the location by calculating the phase difference between the section location and pulse train, but this method requires measuring the angle among encoder, planet gear, and accelerometer while setting up [36]. Commonly the first method is selected when the health monitoring of the planetary gearbox is in consider before layout of planetary gearbox.

**Figure 2:**
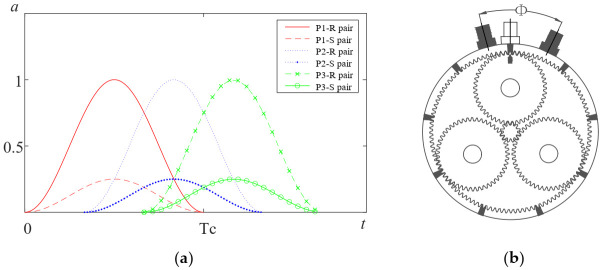
(**a**) Amplitude modulation by time-varying transfer path (**b**) Distribution of fixed fault meshing positions.

To implement the TVTF-VS in practical application, the second method to determine the location of sections is proposed in the following. First of all, the non-hunting ratio between the ring gear and the planet gear should be calculated by the given parameters of the planetary gearbox. As the Figure 2a shows, the signals from different meshing pairs suffers amplitude modulation, and overlap each other. It should be noted that when planet gear $P_{i}$ passes by, the signal perceived by the stationary sensor is dominated by the meshing pairs of the planet gear $P_{i}$, including the planet-ring pair and the planet–sun pair. For a healthy planet gearbox, the maximum peak-to-peak amplitude value in $n_{rest,p}*T_{c}$ is chosen as the center location of the first section. Other sections can be extracted successively by the interval of 2π and the phase difference can be determined by the encoder pulse train.

When there is a localized fault on the planet gear, the meshing points of the fault tooth with the ring gear relative to the ring gear are fixed. The number of fixed fault meshing positions on the ring gear can be calculated by

(3) $$N^{*}=\frac{LCM(Z_{p},Z_{r})}{Z_{p}}$$

For a planet gear with localized fault, the location of the first section can be obtained by moving the sensor. Move the sensor $\Delta \theta =\frac{2\pi }{Z_{r}}$ radian at a time along the axial direction of ring gear for total $\Phi =\frac{2*\pi }{N^{*}}$ radian and collect complete signal every time. The signal set is chosen that has the maximum peak-to-peak amplitude value in $n_{rest,p}*T_{c}$. The position of maximum peak-to-peak amplitude value is chosen as the center location of the first section. The center of the first section represents the moment when the faulty planet gear is just under the sensor and the faulty tooth is exactly in mesh with the ring gear. Other sections can be extracted successively by the interval of 2π.

Taking the type 2 planetary gearbox given in Table 1, for example, the number of fixed fault meshing positions $N^{*}=\frac{LCM(48,108)}{48}=9$ and the distribution of fixed fault meshing positions is shown in Figure 2b. With the sensor moving along the axial direction of ring gear for $\Phi =\frac{2*\pi }{N^{*}}=\frac{2\pi }{9}$ radian, there must be a fixed fault meshing position under the sensor.

The length of one section is then given by

(4) $$\gamma_{sec}=M_{t}*\gamma_{0}$$

where $M_{t}$ donates number of meshing teeth extracted in one section, $\gamma_{0}$ donates length of meshing signal for single tooth. The number of meshing teeth extracted in one section $M_{t}$ is decided by

(5) $$M_{t}=\frac{Z_{p}}{n_{rest,p}}$$

where $Z_{p}$ is the tooth number of planet, $n_{rest,p}$ is calculated by Equation (1), and the length of meshing signal for single tooth $\gamma_{0}$ is given by

(6) $$\gamma_{0}=\frac{f_{s}}{f_{c}*Z_{r}}$$

where $f_{s}$ is the sampling frequency, $f_{c}$ is the rotation frequency of carrier, and $Z_{r}$ is the tooth number of the ring gear.

## 3. Time-Varying Transfer Function

### 3.1. Analysis of Time-Varying Transfer Path

The vibration signal collected from the planetary gearbox has unique characteristics distinguished from that from the fixed axis gearboxes. Multiple gear pairs simultaneous meshing coupling, multiple vibration transfer paths, and time-varying transfer path are the main features of planetary gearbox, which causes complicated amplitude and phase modulation. Summarizing the characteristics of the planetary gearbox can help us better make use of the vibration signal for analysis and diagnosis.

As is well known, the structure of a planetary gearbox is much more complicated than a fixed-axis gearbox. Different transmission ratios could be achieved by fixing any one of the ring gear, sun gear, or carrier. Generally, the ring gear is stationary while the sun gear and the planet gears are rotating as input or output. Only this case is discussed in this paper.

As the planet gear revolves around the sun gear, it also makes self-spinning motion at the same time. The planet gear meshes with the sun gear and ring gear simultaneously and there are a couple of planet gears in one planetary gearbox. Therefore, multiple gear pairs simultaneous meshing produces multiple vibration sources. The vibration signals coupling also increases the difficulties for vibration analysis.

**Figure 3:**
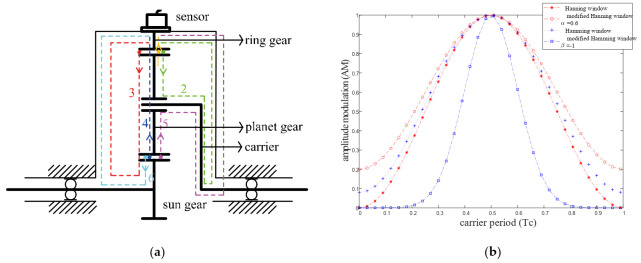
(**a**) Schematic diagram of vibration transfer paths in planetary gearbox (**b**) Modulation effect of different window function.

Figure 3a shows the vibration transfer paths in the planetary gearbox. As Figure 3a shows, there are two gear pairs for one planet gear, and there are three vibration transfer paths for each gear pair. The transfer paths are introduced simply. For planet-ring gear pair: (1) path 1: meshing point-ring gear-sensor; (2) path 2: meshing point-planet gear-planet bearing-carrier-carrier shaft holding bearing-housing-sensor; and (3) path 3: meshing point-planet gear-sun gear-sun gear shaft holding bearing-housing-sensor. For planet-sun gear pair: (1) path 4: meshing point-planet gear-ring gear-sensor; (2) path 5: meshing point-planet gear-planet bearing-carrier-carrier shaft holding bearing-housing-sensor; and (3) path 6: meshing point-sun gear-sun gear shaft holding bearing-housing-sensor. Besides the six paths mentioned above, there exists other vibration transfer paths including longer transfer paths or complicated transfer paths such as the lubricating oil, because the vibration signal is attenuated sharply and vibration going through these transfer paths are neglected.

Assuming that the gears are isotropic, the length of path 2, path 3, path 5, and path 6 are all constant. The constant transfer path leads to constant energy loss, and hence the signals transmitted through these paths are time-invariant. However, the length of path 1 and path 4 are changed with the carrier rotation; therefore, the vibration signals transmitted through time-varying transfer paths are time-variant. This phenomenon leads to complicated amplitude modulation and phase shift. Hence, conventional vibration analysis methods, such as synchronous averaging (SA), cannot be applied to the planetary gearbox directly.

### 3.2. Expression of Time-Varying Transfer Function

As the planet gear approaches the sensor, the signal perceived by the fixed sensor increases and the signal reaches the maximum when the planet gear is just below the sensor. Vice versa, as the planet gear goes away from the sensor, the signal perceived by the fixed sensor decreases and the signal reaches the minimum when the planet gear is opposite the sensor position along the ring gear. The signal perceived by the fixed sensor is the superposition of signals transmitting through all the paths. As a result, the amplitude of the signal perceived by the sensor corresponds to the periodic change in the time-varying transfer path length due to the carrier rotation. Inalpolat [13] initially used the Hanning function to represent the time-varying path modulation effect. The Hanning function assumes that the meshing signal decreases to zero when it has arrived the farthest position from the sensor. Assuming that the meshing vibration signal could still be picked up by sensor even at the farthest position from the sensor, the amplitude modulation phenomenon can be represented by a modified Hanning function:

(7) $$w_{i}=\alpha -(1-\alpha )\cos(\omega_{c}t+\varphi_{n})$$

where α controls the minimum and maximum value and bandwidth of the Hanning window function with 0.5≤α<1; $\omega_{c}$ is the angular velocity of carrier; and $\varphi_{n}$ is initial phase of the planet gear.

For a specific planetary gearbox, when the planet gear goes away from the sensor, the perceived signal by the sensor from the meshing point may attenuate to zero before arriving the farthest position. Based on this point, Liang et al. [37] proposed a modified Hamming function to represent the amplitude modulation phenomenon:

(8) $$w_{i}=e^{\beta {\left(\mathrm{mod}(w_{c}t+\varphi_{n},2\pi )-\pi \right)}^{2}}(0.54-0.46\cos(w_{c}t+\varphi_{n}))$$

where β controls the minimum and maximum value and bandwidth of the Hamming window function with β<0; $\omega_{c}$ is the angular velocity of carrier; and $\varphi_{n}$ is initial phase of the planet gear.

As the Figure 3b shows, different window functions are modeled to represent the amplitude modulation effect of the time-varying transfer path. The key parameters control the bandwidth and the extreme value of the window function. The window function is chosen according to the modulation effect determined by the properties of specific planetary gearbox, such as size, bearing model, ring gear-casing interface, and ring gear flexibility [37]. In order to adapt the window function to simulating more situations, in this paper, amplitude modulation phenomenon is modeled by TVTF:

(9) $$w_{i}=e^{\beta {\left(\mathrm{mod}(w_{c}t+\varphi_{n},2\pi )-\pi \right)}^{2}}(\alpha -(1-\alpha ))\cos(w_{c}t+\varphi_{n}))$$

the meanings of letters in Equation (9) are as same as these in Equations (7) and (8). Equation (9) includes 2 main parameters α and β to controls the minimum and maximum value and bandwidth of the window function. When β=0, Equation (9) is a modified Hanning window and when α=0.54, Equation (9) is a modified Hamming window. Compared with Equations (7) and (8), Equation (9) is more flexible and has higher ability for a generalized description. Thus, Equation (9) can represent the TVTF of a random planetary gearbox. From the point of view of signal processing and mathematics, amplitude modulation corresponds to the multiplication of two signals in a time domain. Hence, when the meshing signal is modulated by the time-varying transfer path, the signal perceived by the transducer is the multiplication of the meshing signal and the corresponding TVTF.

### 3.3. Evaluation Indicator

In order to evaluate the compensation effect of the target planet gear quantitatively and to seek the optimum parameters of the TVTF, an evaluation indicator is desired. Consider the classic damage detection metric Kurtosis [38] given as

(10) $$Kurtosis=\frac{\frac{1}{N}\sum_{i=1}^{N}(x_{i}-\bar{x}{)}^{4}}{{\left[\frac{1}{N}\sum_{i=1}^{N}{\left(x_{i}-\bar{x}\right)}^{2}\right]}^{2}}$$

where N is the total number of data points in the time signal, $x_{i}$ is the *i*th data point, $\bar{x}$ is the mean of the signal. Kurtosis is used to provide a measurement of the peakedness of the signal, i.e., number and amplitude of peaks. On this basis, this paper proposed a new metric called *ND*6, using the sixth moment normalized by the variance to the third power:

(11) $$ND6=\frac{\frac{1}{N}\sum_{i=1}^{N}(x_{i}-\bar{x}{)}^{6}}{{\left[\frac{1}{N}\sum_{i=1}^{N}{\left(x_{i}-\bar{x}\right)}^{2}\right]}^{3}}$$

where *N* is the total number of data points in the time signal, $x_{i}$ is the *i*th data point, and $\bar{x}$ is the mean of the signal. *ND*6 is non-dimensional and more sensitive to the number and amplitude of peaks present in the signal due to the use of the sixth moment. Higher moment corresponds to higher sensitivity. It should be noted that a sharp increasing of sensitivity to the peaks is not desired because it may trigger a false alarm. The parameter of α and β is optimized by interaction to reach the minimum value of *ND*6.

### 3.4. Construction of the Time-Varying Transfer Function

**Figure 4:**
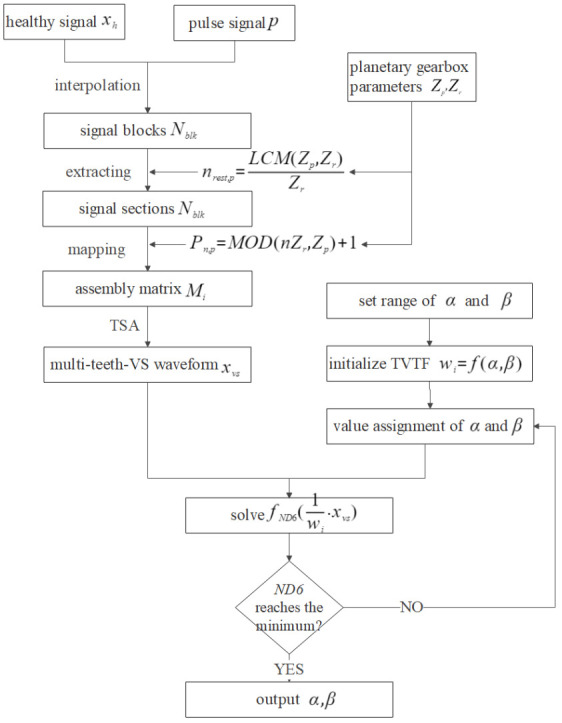
Flow chart of construction of the TVTF.

The TVTF is used to compensate the multi-teeth-VS waveform and the construction process of the TVTF is illustrated in Figure 4. Healthy vibration signal $x_{h}$ and encoder pulse train p are collected simultaneously. Then, the $x_{h}$ is interpolated into individual cycles and $N_{blk}$ signal blocks are obtained. The sections extracted from blocks are mapping to the assembly matrix $M_{i}$ according to $P_{n,p}$. After the TSA procedure, the multi-teeth-VS waveform $x_{vs}$ is obtained. Initialize the TVTF $w_{i}=f(\alpha ,\beta )$, and set range of α and β. The iteration algorithm’s objective is to search appropriate the TVTF, which makes *ND*6 of $\frac{1}{w_{i}}\cdot x_{vs}$ minimum. Finally, the values of α and β are obtained and the TVTF is established. It is noted that different amplitude modulation effects occur in different planetary gearbox, and hence the resultant TVTF is only built for the specific gearbox.

## 4. Multi-Teeth VS Compensated by the TVTF

### 4.1. Technical Route of Fault Detecting by the TVTF-VS

**Figure 5:**
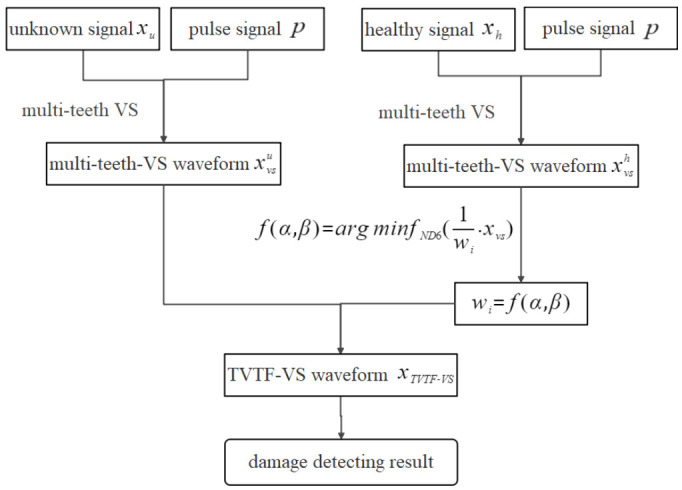
Technical route of fault detecting by the TVTF-VS.

In order to apply the VS to a non-hunting tooth planetary gearbox and diagnose the planetary gearbox effectively, the TVTS-VS is proposed in this paper. Monitoring signal is first processed by multi-teeth VS to get a multi-teeth-VS waveform in spite of a non-hunting ratio, then the TVTF is used to eliminate the amplitude modulation effect so that the incipient fault on the planet gear can be highlighted accurately. The technical route of fault detecting by the TVTF-VS is displayed in Figure 5.

As previously illustrated, the proposed the TVTF-VS methodology can be formulated by:

(12) Xp(t)=1wi1MeMt∑n=0MeKnRest,g−1[Rjx(t)u(t)v(t−nTc)]∗g(t)

where $X_{p}(t)$ is the final signal of interest planet gear after the TVTF-VS methodology, $w_{i}$ is the constructed TVTF, $M_{e}$ denotes the average number for TSA, $M_{t}$ represents the number of the tooth mesh period, which should be multi-teeth sectioning calculated by $M_{t}=\frac{Z_{p}}{n_{Rest,p}}$, $Kn_{rest,g}$ denotes length of the synthesized signal with K complete revolutions of the interesting gear, K is an integer number, $R_{j}$ is a rectangular function with width of $M_{t}$ tooth mesh periods, u(t) is a sampling function with a period of $\Delta t=1/f_{s}$, $v(t-nT_{c})$ is the sampling function centered at time $t=T_{c}$, and g(t) is a sampling function with a period of T(g).

### 4.2. Effectiveness Analysis of TVTF-VS

**Figure 6:**
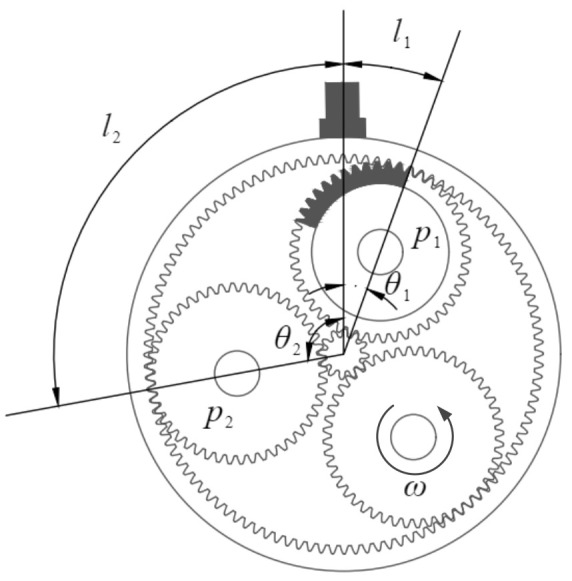
Objective planet gear is at the boundary of capturing of sensor.

To implement the TVTF-VS methodology, the extracted multi-teeth sections should be dominated by the objective planet-ring pair. That is to say, the signal perceived by the sensor is dominated by the signal transmitted from path 1, as depicted in Figure 3a. Figure 6 shows the extreme case where the objective planet gear is at the boundary of the extracted multi-teeth section. The meshing signal of black teeth on the planet gear is included in the extracted signal section. Define the ξ as ratio between arc $l_{1}$ and arc $l_{2}$, and we can obtain:

(13) $$\xi =\frac{l_{2}}{l_{1}}=\frac{\theta_{2}\cdot R}{\theta_{1}\cdot R}=\frac{\theta_{2}}{\theta_{1}}$$

In addition, the angle $\theta_{1}$ and $\theta_{2}$ can be calculated by:

(14) $$\left\{\begin{array}{c} \theta_{1}=\frac{M_{t}}{Z_{r}}\pi \\ \theta_{2}=\frac{2\pi }{N}-\theta_{1} \end{array}\right.$$

Taking Equation (14) into the Equation (13), we can obtain:

(15) $$\xi =\frac{2Z_{r}-N\cdot M_{t}}{N\cdot M_{t}}$$

where $Z_{r}$ is tooth number of ring gear, N is number of planet gear, and $M_{t}$ is number of meshing teeth extracted in one section. It should be noted that the TVTF-VS methodology is effective as long as ξ>1 theoretically. However, there exists a lot of background noise for in situ application.

In order to reduce interference from other meshing pairs, a large ratio ξ is expected. Hence, the bigger ξ is, the better TVTF-VS performs. In the future, a more accurate TVTF should be constructed to model the time-vary transfer path. We aim to reduce the interference from other meshing pairs and further improve the effect of diagnosis and prognosis.

## 5. Experimental Validation

In this section, the effectiveness of the proposed TVTF-VS methodology is evaluated by experimental data. Details of experiment and discussion of results are as follows.

### 5.1. Experimental Test Rig

**Figure 7:**
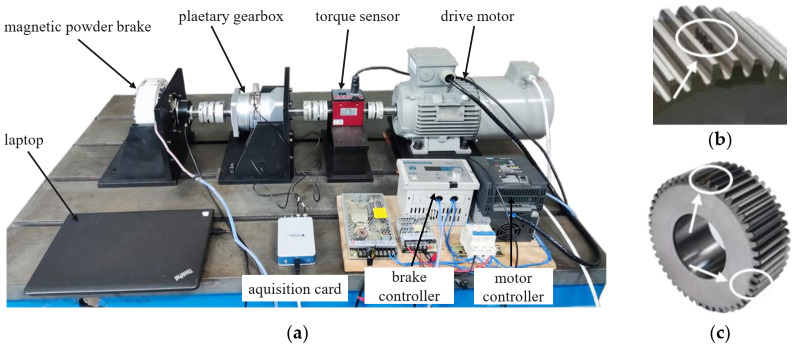
(**a**) Experimental test rig, (**b**) pitting planet gear, and (**c**) spalling and root crack planet gear.

Figure 7a shows the planetary gearbox test rig used for data collection. This test rig contains a 3 kw, 0–3000 rpm variable speed AC drive motor, a 0–20 NM dynamic torque sensor, a planetary gearbox, a 50 ppr encoder, and a 0–80 NM magnetic powder brake. Key parameters of planetary gearbox are listed as type 2 in Table 1. The encoder is installed on the output shaft of the planetary gearbox and generates 50 pulse every one carrier rotation. The encoder pulse train is used to calculate the rotation speed and serve as keyphasor in interpolation procedure.

Vibration signals under healthy condition and two faulty conditions are collected, respectively. In the two faulty conditions, one is the planet gear with intentionally created pitting using laser machining (LM). The pitting is processed into irregularly distributed points to simulate actual working conditions. The other is the planet gear with a compound fault containing both spalling and a root crack created by electro discharge machining (EDM). Spalling is about 25% of tooth width and the root crack is 25% depth closed when in mesh with ring gear. The two faulty planet gears are shown in Figure 7b,c.

An accelerometer was mounted on the external of ring gear. It should be mentioned that the encoder pulse train and vibration are picked up simultaneously by an acquisition card (NI 9234) with the sampling of 25.6k HZ. Experiments were carried out under different working conditions.

### 5.2. Baseline

**Figure 8:**
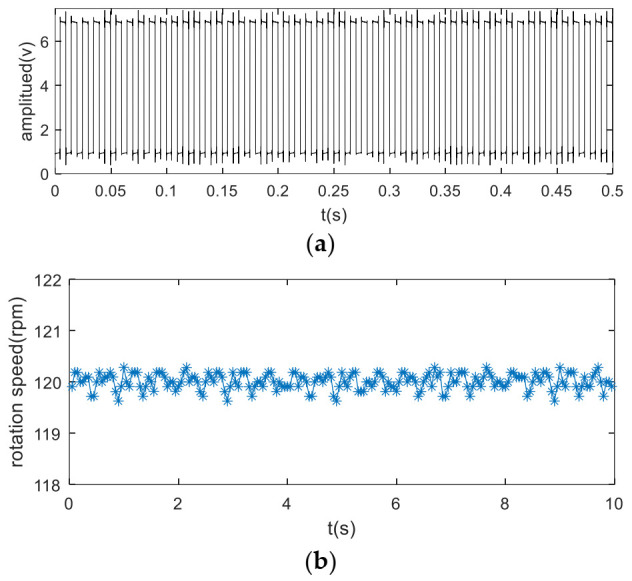
Waveform plots: (**a**) encoder pulse train (**b**) shaft speed plot.

**Figure 9:**
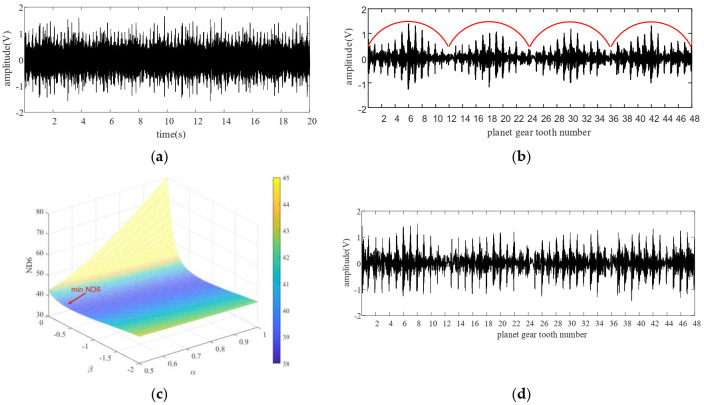
Baseline signal with 1200 rpm analysis results: (**a**) vibration signal, (**b**) multi-teeth VS waveform, (**c**) the ND6 with different value of α and β, and (**d**) the TVTF-VS waveform.

**Figure 10:**
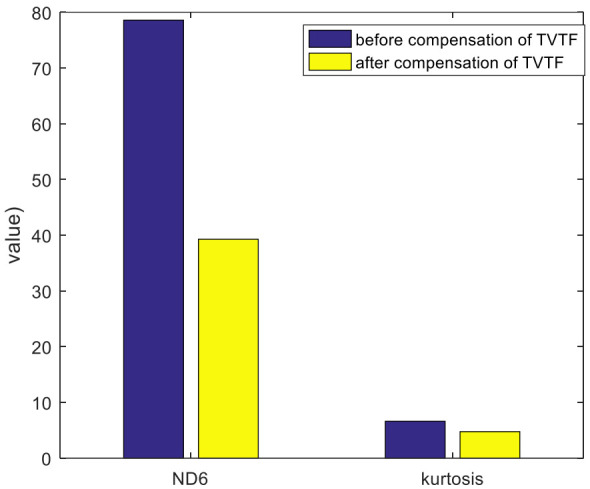
Kurtosis and *ND6* value before and after compensation of the TVTF.

The baseline signal is used to construct the TVTF and to verify the feasibility of proposed methodology preliminarily. Collected encoder pulse train and corresponding speed plots calculated by the intervals of the pulse train are shown in Figure 8a,b. Figure 9a shows the waveform of baseline signal. The baseline signal is interpolated by the pulse train, and the multi-teeth signal sections are extracted. The extracted sections are mapped into an assembly matrix and combined together as an assembly waveform. After 10 times of TSA, the multi-teeth VS waveform of a planet gear is shown in Figure 9b. The amplitude modulation can be observed clearly, and hence the local fault can be masked easily. The TVTF in Equation (9) is chosen. As Figure 9c shows, the condition indicator ND6 reaches the minimum value 39.09 when α=0.5,β=−0.45. The TVTF-VS waveform after the TVTF compensating is shown in Figure 9d. Compared with multi-teeth-VS waveform, the signal of resultant TVTF-VS waveform is less modulated by the transfer path. The TVTF is then used to compensate the modulated signal in the following fault detection of unknown signals. Figure 10 shows the kurtosis and ND6 value before and after compensation of TVTF. The ND6 can effectively shows the decreasing tendency of peakedness and is more sensitive to the variation than the kurtosis.

In the case of heathy planet gear, even though TVTF-VS waveform is compensated by the TVTF, the amplitude of signal near the connections of multi-teeth-sections is faint, such as tooth number 12, 24, and 36 in Figure 9d. This is most likely due to the approaching of other gear pairs and vibration of the objective planet-ring pair is interfered. Additionally, inevitable manufacturing and assembling errors also add to the distortion of reconstructed time-domain signal.

**Figure 11:**
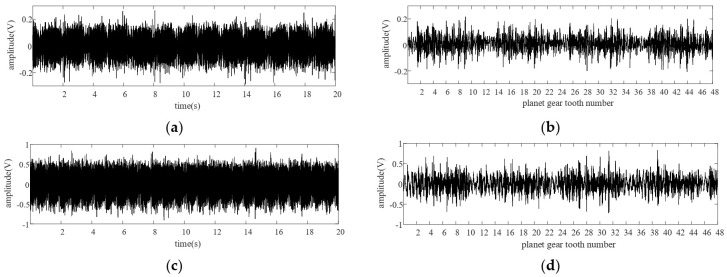
Vibration signal and the resultant TVTF-VS waveform: (**a**) vibration signal under 400 rpm, (**b**) TVTF-VS waveform under 400 rpm, (**c**) vibration signal under 800 rpm, (**d**) TVTF-VS waveform under 800 rpm.

**Figure 12:**
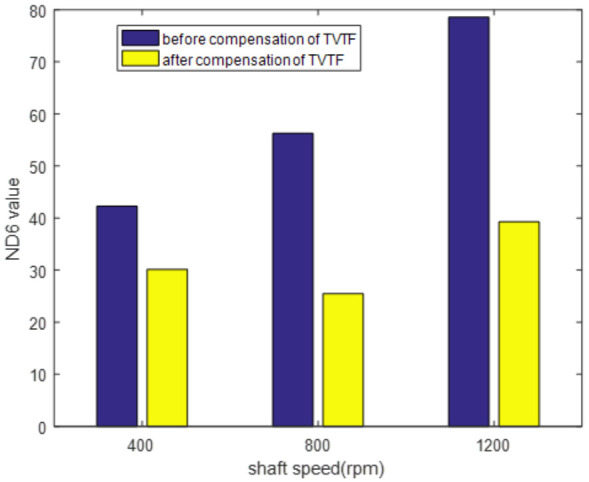
*ND*6 value before and after compensation of TVTF with different speeds.

In order to verify effectiveness of the TVTF-VS under different speeds, three baseline experiments are conducted with the speed of 400 rpm, 800 rpm, and 1200 rpm, respectively. The vibration signal and resultant TVTF-VS waveform under 400 rpm and 800 rpm are given in Figure 11. Results show that TVTF-VS is valid under different speed and it can extract the waveform of a planet gear successfully. Figure 12 is the ND6 value before and after compensation of TVTF with different speeds. All TVTF-VS waveforms are obtained using the same TVTF constructed by vibration signal under 1200 rpm. ND6 value decreases obviously after compensation by TVTF. It is shown that the TVTF is universal for one planetary gearbox regardless of different working conditions. In addition, Figure 12 also shows that the compensation effect decreases with increasing speed due to higher noise. At last, we chose the condition where the motor speed was set to be 1200 RPM for the following experiments.

### 5.3. Planet Gear Pitting Detection

**Figure 13:**
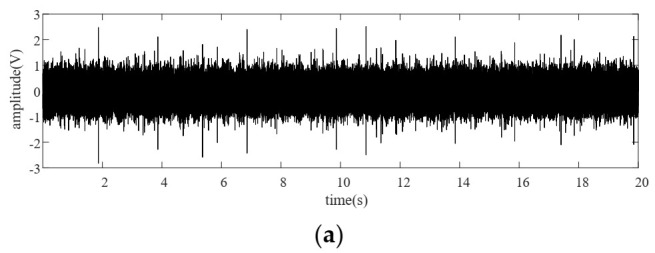

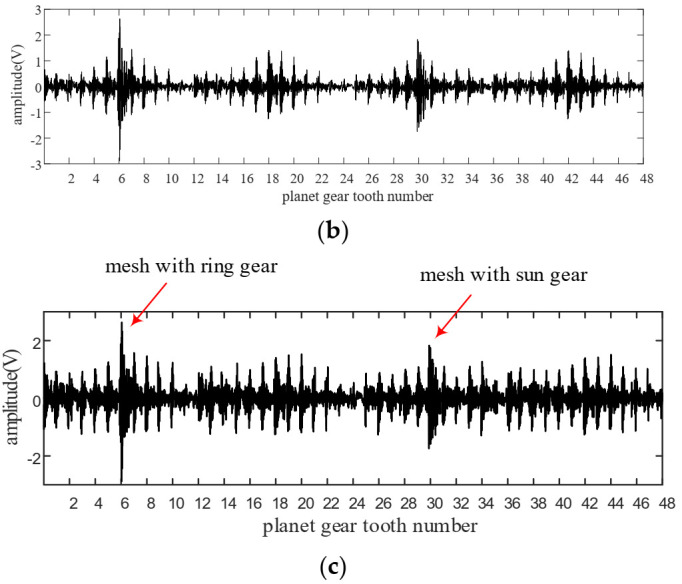
Signal analysis results with pitting fault on planet gear: (**a**) vibration signal, (**b**) multi-teeth VS waveform, (**c**) TVTF-VS waveform.

Figure 13a shows the vibration signal from a planetary gearbox with a pitting planet gear. Comparing Figure 13a with Figure 9a, we cannot identify the fault symptoms visually due to the complexity of modulation. The weak impacts induced by the incipient pitting tooth are submerged in the vibration signal and background noise. Figure 13b presents the multi-teeth-VS waveform. As the figure shows, amplitude modulation can be recognized clearly. In order to amplify the signal characteristic when the planet gear is going far away from the sensor position, the TVTF is applied to compensate the multi-teeth-VS waveform. Figure 13c is the resultant TVTF-VS waveform after applying the proposed methodology. As Figure 13c shows, there is an evident impact at tooth number 6, which implies a local fault. As for the fluctuation at tooth number 30, it is because the pitting tooth is just in mesh with the sun gear aligning to the sensor. When there is an interval of half the number of teeth of the planet gear between abnormal impulses, only one localized fault should be considered firstly. It is because the impulse is generated when the faulty tooth is in mesh with the ring gear or the sun gear successively. When the faulty tooth is in mesh with the sun gear, the transmission path is longer and the vibration signal suffers more energy loss. Hence, the amplitude of impulse induced by engagement with the sun gear is smaller.

In Figure 13c, the distortions can be recognized at the boundary of extracted sections in the TVTF-VS waveform such as tooth number 12, 24, 36, and 48. The boundary is corresponding to the moment when the planet gear goes away from the sensor. At this moment, the time-varying transfer path is farthest in the extracted signal, and the vibration signals are attenuated greatly during the transmission. Moreover, the other planet gears are getting closer to the sensor at that time. As a result, the signal perceived by the sensor is interfered and overlapped by the meshing signal coming from the other meshing pairs.

### 5.4. Compound Localized Faults Location

**Figure 14:**
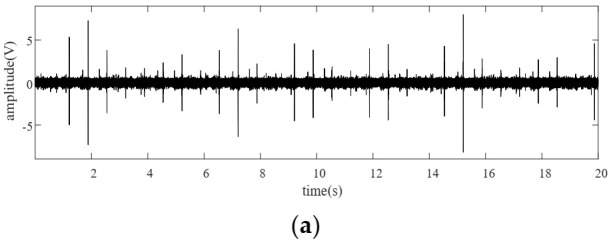

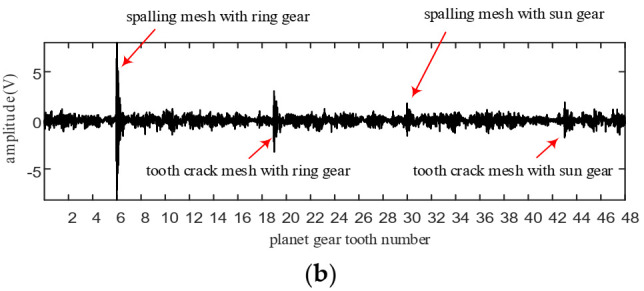
Signal analysis results with compound localized faults on planet gear: (**a**) vibration signal with compound localized faults, (**b**) TVTF-VS waveform.

In order to test TVTF-VS’s capability of fault diagnosis in case of multiple faults, compound localized faults experiment are conducted. Figure 14a presents the vibration signal of the planetary gearbox with spalling and pitting faults on the planet gear. After applying the TVTF-VS, the resultant TVTF-VS waveform is shown in Figure 14b. Although distinct impacts can be identified in Figure 14a, some concrete details about the faulty gear are unknown. It is necessary to estimate the severity of the damaged gear and further analyze the reasons causing the faults. After applying the TVTF-VS methodology to the signal, the TVTF-VS waveform is obtained. As shown in Figure 14b, the clear fault symptoms are distinguished at tooth number 6, 19, 30, and 43. The quantity and relative location of the local faults then can be determined. It should be noted that the impact induced by the planet gear fault is more discernable when in mesh with the ring gear than in mesh with the sun gear. Then, it can be deduced that the impact at tooth number 30 is induced by tooth number 6 when in mesh with the sun gear. Similarly, the impact at tooth number 43 is induced by tooth number 19 when in mesh with the sun gear. The fact is that there is tooth spalling at tooth number 6 and root crack at tooth number 19. Although fault type cannot be determined from the TVTF-VS waveform, damage severity of localized faults could be evaluated by the amplitude of the impulses. In Figure 14, the amplitude of the impulse at tooth number 6 is most distinct while the amplitudes of other impulses are relatively small. Thus, it can be concluded that localized fault at tooth number 6 is more severe and may cause many more negative effects. In practical application, fault location information and damage condition provide more reference information for maintenance staff. It should be noted that this research is focused on the detection and location of local faults, and their classification is out of the scope of this research.

## 6. Conclusions

This paper presents a planet gear fault diagnosis methodology called vibration separation compensated by a time-varying transfer function (TVTF-VS), which makes the VS further developed in diagnosis of non-hunting tooth planetary gearboxes. Based on VS, multi-teeth VS is proposed to extract the meshing signal of a planet gear. Then, a parameter optimization algorithm is developed to construct the TVTF using a segment of healthy signal. The TVTF is used to overcome the amplitude modulation effect caused by planetary movements, and highlight weak characteristics induced by incipient local fault. Finally, the localized fault detection conclusions are drawn using the resultant waveform. A distinct advantage of the TVTF-VS is that only one vibration transducer is needed. Two sets of planet gear fault-seeded experiments with pitting and compound faults are conducted to verify the proposed methodology. Experimental results show that the proposed method can effectively detect and locate local faults on the planet gear and provide more details about the faulty planet gear in non-hunting tooth planetary gearboxes. Damage condition and location information of faults could be obtained by the TVTF-VS waveform. While applying the process of the proposed method, selection of the TVTF determines the compensation effect and diagnosis result. In the future, a more accurate TVTF should be modeled in order to match planetary gearboxes with different specifications.

## Figures and Tables

**Table 1:** Parameters of planetary gearbox.

	Sun Gear $\left(Z_{s}\right)$	Planet Gear $\left(Z_{p}\right)$	Ring Gear $\left(Z_{r}\right)$	Number of Planets (N)
**Type 1**	25	22	71	3
**Type 2**	12	48	108	3

**Table 2:** Meshing sequence of planet gear for planetary gearbox 1.

***n***	0	1	2	3	4	5	6	7	8	9	10
$P_{n,p}$	1	6	11	16	21	4	9	14	19	2	7
***n***	11	12	13	14	15	16	17	18	19	20	21
$P_{n,p}$	12	17	22	5	10	15	20	3	8	13	18

**Table 3:** Meshing sequence of planet gear for planetary gearbox 2.

***n***	0	1	2	3	4	5	6	7	8	9	10	11
$P_{n,p}$	1	13	25	37	1	13	25	37	1	13	25	37

## Data Availability

Data sharing is not applicable to this article.
